# Comparison of Physical Adsorption and Covalent Coupling Methods for Surface Density-Dependent Orientation of Antibody on Silicon

**DOI:** 10.3390/molecules27123672

**Published:** 2022-06-07

**Authors:** Katarzyna Gajos, Panagiota Petrou, Andrzej Budkowski

**Affiliations:** 1 Smoluchowski Institute of Physics, Jagiellonian University, Łojasiewicza 11, 30-348 Kraków, Poland; andrzej.budkowski@uj.edu.pl; 2 Institute of Nuclear & Radiological Sciences & Technology, Energy & Safety, NCSR Demokritos, 15341 Athens, Greece; ypetrou@rrp.demokritos.gr

**Keywords:** antibody orientation, antibody immobilization, random sequential adsorption, TOF-SIMS

## Abstract

The orientation of antibodies, employed as capture molecules on biosensors, determines biorecognition efficiency and bioassay performance. In a previous publication we demonstrated for antibodies attached covalently to silicon that an increase in their surface amount Γ, evaluated with ellipsometry, induces changes in their orientation, which is traced directly using Time-of-Flight Secondary Ion Mass Spectroscopy combined with Principal Component Analysis. Here, we extend the above studies to antibodies adsorbed physically on a 3-aminopropyltriethoxysilane (APTES) monolayer. Antibodies physisorbed on APTES (0 ≤ Γ ≤ 3.5 mg/m^2^) reveal the Γ ranges for flat-on, side-on, and vertical orientation consistent with random molecular packing. The relation between orientation and Γ is juxtaposed for silicon functionalized with APTES, APTES modified with glutaraldehyde (APTES/GA) and N-hydroxysuccinimide-silane (NHS-silane). Antibody reorientation occurs at lower Γ values when physisorption (APTES) is involved rather than chemisorption (APTES/GA, NHS-silane). At high Γ values, comparable proportions of molecules adapting head-on and tail-on vertical alignment are concluded for APTES and the NHS-silane monolayer, and they are related to intermolecular dipole–dipole interactions. Intermolecular forces seem to be less decisive than covalent binding for antibodies on the APTES/GA surface, with dominant head-on orientation. Independently, the impact of glutaraldehyde activation of APTES on vertical orientation is confirmed by separate TOF-SIMS measurements.

## 1. Introduction

The immobilization of antibodies onto surfaces, to act as detection molecules in immunosensors, requires special attention due to the dependence between the various possible antibody orientations and its binding activity [1,2]. Immunoglobulin G (IgG) is a Y-shaped molecule with an Fc trunk and two antigen binding sites located on the two Fab arms that can adapt on the surface four different orientations varying in accessibility of binding sites: flat-on, site-on, head-on and tail-on [2]. As a consequence, the orientation of the surface-immobilized antibodies is crucial for the analyte binding efficiency of the immunosensor surface. Although various affinity coupling methods for IgG were established [3], random immobilization by physical adsorption or covalent binding through the amine group on the protein molecules is still widely applied due to its fast and simple procedure [3]. To enhance antibody adherence to silicon or gold modified with self-assembled monolayers (SAM), physical adsorption is commonly supplemented with covalent grafting, when the SAM surfaces are activated with chemical linkers such as glutaraldehyde (GA) for amino-terminated monolayers [4,5,6,7]. However, this additional step of the immobilization protocol might modify the orientation of IgG, as indicated for the activation of carboxyl-terminated SAM with N-hydroxysuccinimide/1-Ethyl-3-(3-dimethylaminopropyl)carbodiimide (NHS/EDC) [6].

The arrangement of immobilized antibodies is a complex phenomenon that involves molecule–molecule and molecule–surface interactions (reviewed in [8,9,10]). The dominant antibodies orientation is determined by the amount of adsorbed molecules Γ [11,12] as well as by physical (electrostatic, hydrophobic, van der Waals) forces and the reactivity of amine groups on the protein molecule, which depends on immobilization conditions, e.g., the pH of solution [9,13]. It is of practical interest to compare the orientation of antibodies immobilized on amino-terminated SAM with and without GA activation, and to view it in relation to that on the NHS-terminated SAM surface. Since physical adsorption precedes covalent immobilization [2,14], such a comparison could provide some information on the impact on the final arrangement of the immobilized antibody of the molecule–molecule and molecule–surface interactions characteristic of physisorption and chemisorption.

The determination of the orientation of surface-immobilized antibodies is also a challenging issue [2]. Usually, the dominant antibody orientation can be inferred indirectly from the antigen-binding efficiency or from its surface density Γ [2,3]. The surface density Γ specifies the orientation of antibody, as the increase in Γ decreases the surface area accessible to a single molecule, forcing its more vertical alignment. However, the geometric relation between Γ and the IgG orientation [11], commonly applied to deduce the molecular arrangement from the Γ values determined with surface analysis techniques (as reviewed in [3]), cannot resolve the ambiguities of orientations available for the same Γ ranges (e.g., tail-on vs. head-on). In addition, this approach assumes the questioned highly efficient close-packed arrangement of molecules on the surface [15]. Only recently has this issue been experimentally revisited [12] to show that the Γ values corresponding to a particular IgG orientation are significantly lower than those deduced from the above approach.

**Figure 1:**
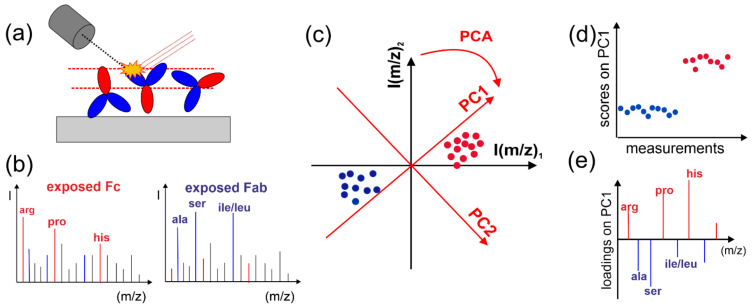
(**a**,**b**) Comparison of the antibody orientation in different samples, based on TOF-SIMS sensitivity of the outermost region of immobilized proteins (marked by red horizontal lines) (**a**) examined with regard to their amino acid composition (**b**), distinct for exposed Fc and Fab domains. (**c**–**e**) PCA examines numerous TOF-SIMS measurements (points) in a multidimensional space with axes spanned by intensities of different mass signals, and it determines a few sequential directions of the greatest uncorrelated variations called Principal Components (PC1, PC2, PC3) (**c**). PCA results are presented as the plots of scores on PCs from different TOF-SIMS measurements, which are separated according to the features maximized by each PC (**d**) and the plots of loadings for PCs from mass signals, which enable the interpretation of PCs (**e**).

In contrast, antibody orientations can be directly deduced from microscopic visualizations of single molecules by AFM [16,17] compared with insights from Monte Carlo and molecular dynamics (MD) simulations [16,18]. In turn, direct analysis of the dominant IgG orientation can be provided by Time-of-Flight Secondary Ion Mass Spectroscopy (TOF-SIMS) combined with Principal Component Analysis (PCA) [3,5,6,8,12,19,20]. While TOF-SIMS examines the outermost region of adsorbed molecules with regard to their amino acid composition, which is different for the exposed Fc and Fab domains (Figure 1a,b), PCA enhances the chemical specificity of TOF-SIMS and enables a comparison of different samples (as explained in Figure 1c–e). TOF-SIMS supported with PCA has been applied to compare the orientations adopted by antibodies immobilized on different surfaces of various types (e.g., gold or silicon modified with SAMs [5,6,20,21], polymer layers [20,22,23] and brushes [24], protein monolayers [21,25]), in order to analyze the factors determining molecular orientation (other than surface amount Γ of molecules) and to correlate them with protein biorecognition (as extensively reviewed in [8,26]). In fact, the above approach provides the relation between different samples of the average composition of the outermost regions of immobilized antibodies expressed by the PCA scores, reflecting the average ratio *f_Fc_* of the exposed area of Fc domain to the footprint area of the entire molecule (that is, the sum of exposed Fc and F(ab)_2_). However, exact values of the *f_Fc_* ratio are not evaluated, and therefore, the question of the proportions of antibodies with head-on and tail-on orientation, coexisting at high surface density Γ, cannot be resolved.

In our recent publication [12], we addressed the issue of surface density Γ-dependent orientation of adsorbed antibodies, with both the changes in Γ and in the orientation determined directly for the first time, using ellipsometry and TOF-SIMS with PCA, respectively. Two covalent methods for coupling IgG to silicon surfaces were analyzed involving surface chemical activation with N-hydroxysuccinimide-silane (NHS-silane) or amino-silane activated with glutaraldehyde (APTES/GA). The Γ ranges determined for subsequent flat-on, side-on, and vertical orientations were consistent with random molecular packing. For vertical orientations, the data together with the insights from MD simulations [16] allowed us to evaluate the proportions of molecules with head-on and tail-on alignment.

Here, we extend the above TOF-SIMS and PCA studies on antibodies chemisorbed on silane-modified surfaces [12] to those adsorbed physically on a surface with a 3-aminopropyltriethoxysilane (APTES) monolayer. First, the impact of glutaraldehyde activation of APTES [2,3,5] on the dominant vertical orientation of IgG (for the highest Γ range) is examined. Second, the antibodies physisorbed on APTES [27] for a wide range of Γ values, corresponding to all possible IgG orientations, are compared using TOF-SIMS and PCA supplemented with ellipsometry. The Γ ranges for flat-on, side-on, and vertical orientations are determined. Third, to evaluate how the immobilization method determines changes in IgG orientation with surface density Γ, the PCA scores for antibodies on APTES, NHS-silane [12] and APTES/GA modified surfaces [12] are compared with insights from MD simulations [16] on the exposure *f_Fc_* of the Fc domain for different IgG orientations (i.e., flat-on, side-on, head-on and tail-on). Since the average *f_Fc_* ratio values are linearly related to the PCA scores, the latter values determined for flat-on and side-on orientations can be used to evaluate, based on the PCA scores, the average *f_Fc_* ratio for the vertical mixed head-on and tail-on orientations. Then, using again the insights from the MD simulation, the proportion of antibodies adapting both vertical orientations can be resolved. Furthermore, since the values of the *f_Fc_* ratio can be related to the PCA scores obtained for different immobilization methods, all these scores can be rescaled, and the relations between orientation and surface density Γ for different surface functionalization schemes can be juxtaposed and compared. This comparison, consistent with the examination of the first issue, differentiates the immobilization methods with respect to the characteristic Γ values for IgG reorientation as well as the proportions of IgG molecules with head-on and tail-on alignment for the highest Γ range. All of these issues are discussed in terms of relevant molecule–molecule and molecule–surface interactions.

## 2. Results and Discussion

### 2.1. Dominant Vertical IgG Orientation: Comparison of the Results of IgG Physisorption on Amino-Silane with Those of Conjugation on Amino-Silane Activated with Glutaraldehyde

**Figure 2:**
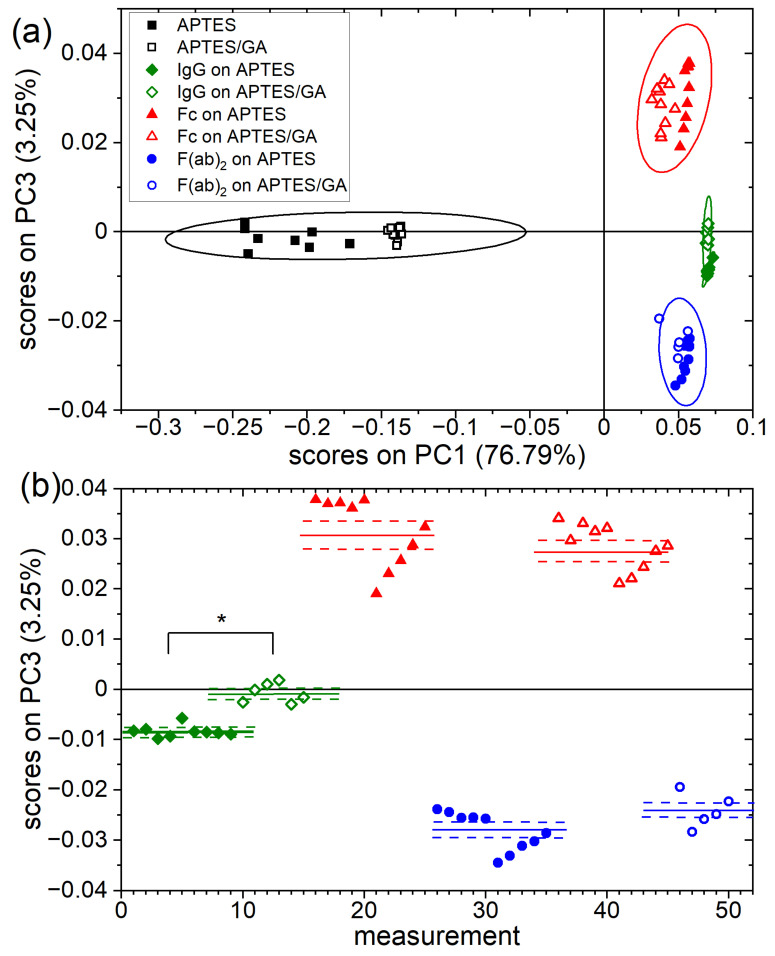
TOF-SIMS and PCA analysis of the orientation of the IgG (goat anti-rabbit IgG antibody) molecules on surfaces modified with APTES and APTES/glutaraldehyde. (**a**) PC1 vs. PC3 scores plot of TOF-SIMS signals recorded for PCA model involving bare substrates, IgG coated substrates, as well as substrates coated with F(ab)_2_ and Fc antibody fragments as reference layers. The ellipses around each of the grouped data points represent the 95% confidence limit. (**b**) The PC3 scores for individual measurements show the difference in the dominant orientation for antibodies immobilized on APTES and APTES/GA-modified silicon surfaces (* significantly different from each other, *p* < 0.05). Solid and dashed lines represent the mean values of the scores on PC3 and the standard deviation of the mean, respectively.

The dominant vertical orientations, adapted for high surface density (Γ > 2.2 mg/m^2^ [12]) by the antibodies physisorbed on amino-silane layers or covalently bound on glutaraldehyde-activated amino-silane layers, are compared by PCA analysis of TOF-SIMS data. For this purpose, layers of goat anti-rabbit IgG antibody (IgG) as well as layers of Fc and F(ab)_2_ fragments of this antibody on APTES and APTES/GA surfaces, together with both bare substrate types, were analyzed with TOF-SIMS. Details on sample preparation and analysis are presented in Section 3. The TOF-SIMS data set, examined with PCA, consists of the relative intensities of 36 ion fragments characteristic for amino acids [28] from 80 spectra. PCA results are represented by the scores plot (PC3 vs. PC1) in Figure 2a. The first principal component PC1, which captures the majority of the variance between samples (76.79%), differentiates bare substrates from protein layers and, therefore, can reflect surface coverage with proteins. While PC2 is not informative, PC3 describes composition changes independent of those represented by PC1 due to the orthogonality of Principal Components. Therefore, PC3, which captures the 3.25% of the variance, distinguishes reference samples covered with Fc or F(ab)_2_ fragments of IgG molecules (Figure 2a). Therefore, for the IgG layers, the scores on PC3 can be regarded as an indicator of the dominant vertical orientation of immobilized antibodies.

The scores on PC3, corresponding to the measurements of the whole IgG molecules and IgG fragments immobilized (Figure 2b), provide insight into antibody orientation. The data points for IgG molecules (green diamonds) are clearly divided into two groups with different mean PC3 scores, as confirmed by the ANOVA test. Furthermore, the different values of PC3 scores corresponding to whole IgG molecules on the APTES/GA and APTES surfaces are shifted toward those representing the Fc and F(ab)_2_ fragments, respectively. These results, obtained for high surface density Γ, indicate differences in the vertical IgG orientations induced by GA activation of APTES that result in a higher fraction of immobilized molecules with inactive head-on rather than active tail-on alignment compared to APTES. This is consistent with the conclusions of Section 2.3.

**Figure 3:**
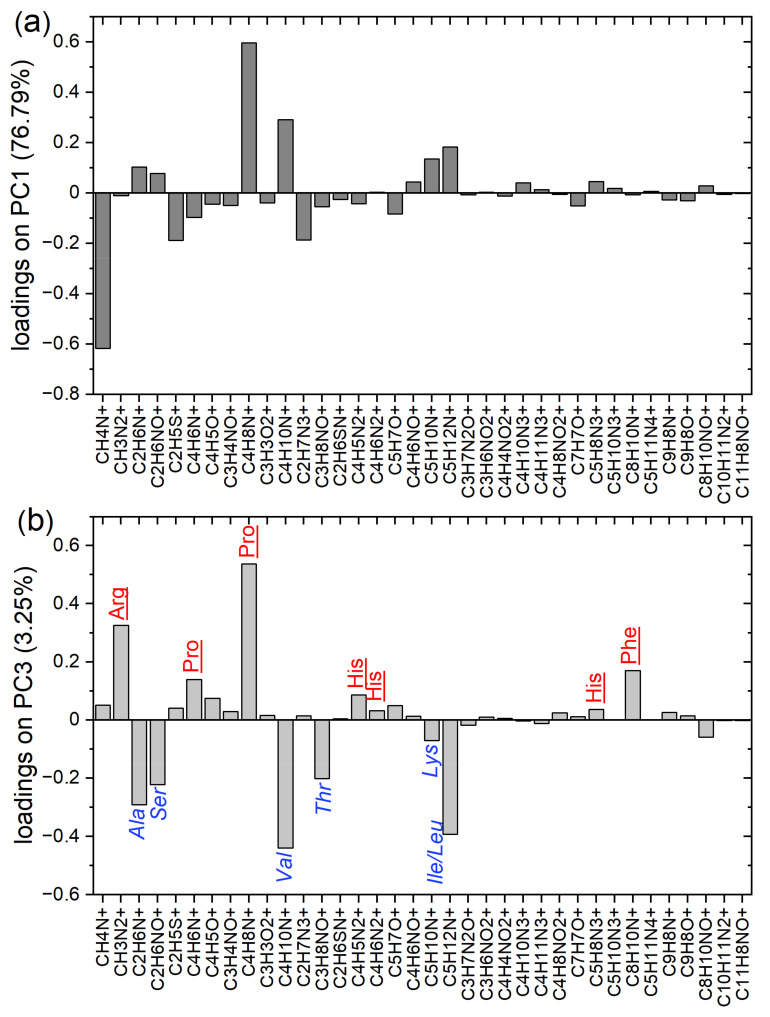
Loadings plots for the principal components presented in Figure 2. (**a**) Loadings plot for PC1. (**b**) Loadings plot for PC3: amino acid ion fragments abundant in the Fc domain (red underlined) load in the positive direction, while amino acid fragments with higher content in the F(ab)_2_ domain (blue italic) load in the negative direction.

The uncorrelated composition changes, attributed above to PC1 and PC3 with the results for the reference samples (bare substrates and substrates modified with IgG fragments), are confirmed by the loadings plots for both Principal Components, as presented in Figure 3. PC1 is negatively loaded mainly by the signals corresponding (in addition to amino acids) to the bare substrate: CH_4_N^+^ derived (also) from APTES and C_2_H_5_S^+^ overlapping with SiO_2_H^+^ from silicon oxide (Figure 3a). On the contrary, the intense signals derived from proteins (C_4_H_8_N^+^, C_4_H_10_N^+^, C_5_H_10_N^+^, C_5_H_12_N^+^ and C_2_H_6_N^+^) load PC1 positively. Therefore, PC1 can reflect the coverage of the substrate with proteins. In turn, PC3 is loaded (Figure 3b) positively and negatively by the ion fragments characteristic for amino acids abundant in the domain Fc (proline, arginine, histidine, phenylalanine) and the domain F(ab)_2_ (serine, alanine, leucine, and threonine), respectively. Hence, PC3 can be related to the orientation of IgG. These results are in agreement with our previous TOF-SIMS studies of the same antibody [12] and with data from the literature on the amino acid composition of other IgG1 molecules [5,20].

### 2.2. Surface Density Dependent Orientation of IgG Molecules Physisorbed on Amino-Silane

**Figure 4:**
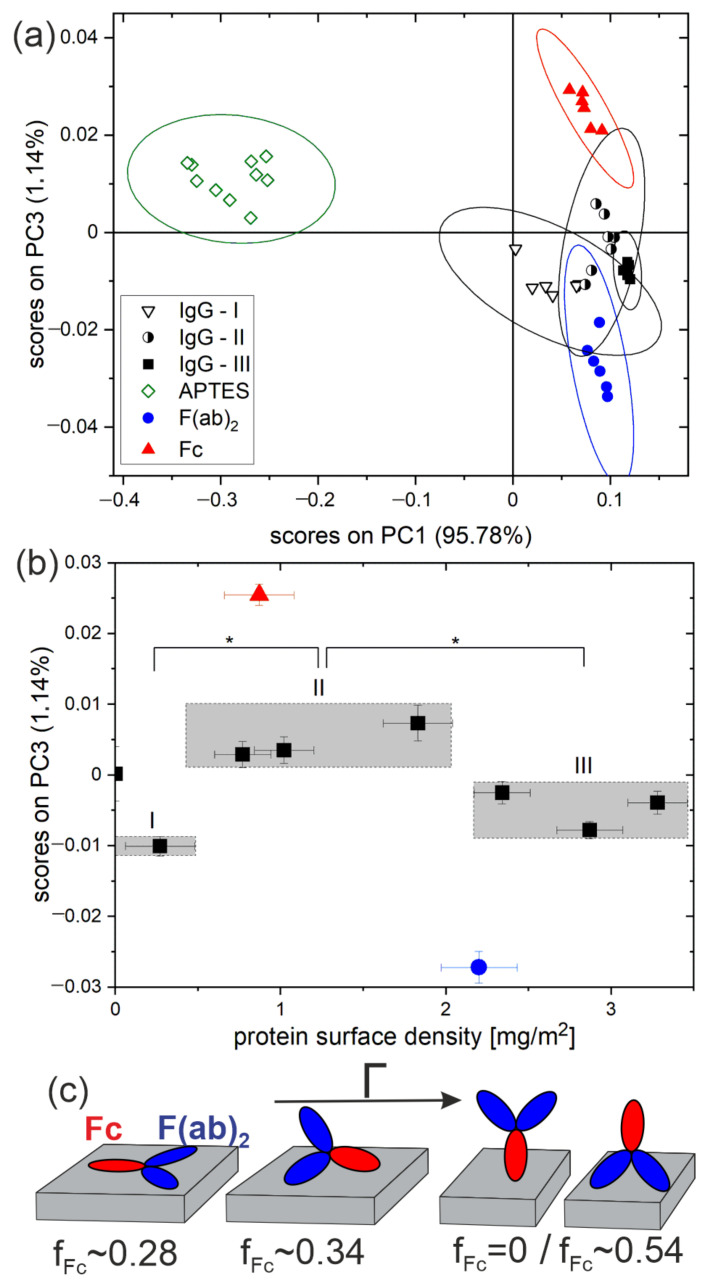
TOF-SIMS and PCA analysis of the surface density-dependent orientation of IgG molecules on APTES-modified silicon substrates. (**a**) PC1 vs. PC3 scores plot for a developed PCA model involving bare substrate, representative immobilized IgG layers with surface densities corresponding to different orientations (I—flat-on, II—side-on and III—vertical orientation), as well as reference surfaces with immobilized antibody fragments F(ab)_2_ and Fc. The ellipses around each of the grouped data points represent the 95% confidence limit. (**b**) Mean values of the scores on PC3 versus the surface density Γ of IgG molecules. Three data points groups corresponding to different IgG orientations (in sequence flat-on, side-on, and tail-on/head-on) can be distinguished (* significantly different from each other, *p* < 0.05). Error bars are standard deviations determined from 6 to 10 TOF-SIMS measurements and 5 ellipsometry measurements of the same sample. (**c**) Scheme of subsequent orientations of immobilized IgG molecules, adapted with increasing protein surface density Γ, with the corresponding values of the areal fraction of the exposed Fc domain (*f_Fc_*) deduced from MD simulations by Vilhena et al. [16].

To analyze the surface density Γ-dependent orientation of antibodies physisorbed on APTES monolayer, a PCA model able to capture the relevant features of TOF-SIMS data characteristic for antibodies with distinct orientations, as well as those of reference samples, must first be constructed [12]. TOF-SIMS spectra were recorded for reference substrates modified with APTES without proteins or coated with the Fc and F(ab)_2_ fragments of goat IgG as well as for substrates modified with APTES coated with whole goat IgG molecules with Γ values corresponding to different orientations [12]: (i) flat-on, (ii) side-on, and (iii) vertical alignment. Such samples were obtained using the IgG solutions with concentrations adjusted according to a previously determined adsorption isotherm (Section 3 and Figure S1 in the Supplementary Materials). Then, a PCA model was developed for the data set based on 40 recorded TOF-SIMS spectra but taking into account only the relative intensities of the ion fragments characteristic for amino acids (listed in Figure S2, the same as in [12]). The obtained PCA model is represented by the PC3 vs. PC1 scores plot (Figure 4a) and respective loadings plots (Figure S2 in the Supplementary Materials). Due to the contributions of signals from the substrate to the signals characteristic for amino acids, PC1 that captures the majority of the variance (95.78%) is related to the coverage of the substrate with proteins. While PC2 is not informative, PC3 (capturing 1.14% of the variance) describes uncorrelated composition changes reflecting immobilized IgG orientation. This can be concluded based on the separation on the PC3 axis of the projections of data points corresponding to reference samples coated with the fragments Fc and F(ab)_2_ (Figure 4a). In addition, this can be confirmed by the positive and negative loadings on PC3 of the ion fragments of amino acids abundant in the Fc and F(ab)_2_ domains, respectively (Figure S2). These loadings are in agreement with those for the same antibody immobilized by chemisorption [12].

Subsequently, the TOF-SIMS data recorded for all examined APTES surfaces with different Γ amounts of immobilized antibodies (0 ≤ Γ ≤ 3.5 mg/m^2^) were projected onto the developed PCA model to provide the mean values of PC3 scores that are plotted as a function of surface density Γ (Figure 4b). Each point in this plot represents one sample, with Γ determined as an average from a set of spectroscopic ellipsometry measurements, and the mean value of PC3 scores determined from a set of recorded TOF-SIMS spectra. Since the scores values on PC3 differentiate between the compositions characteristic of the Fc and F(ab)_2_ fragments, they also characterize the antibody orientation, which is reflected by the ratio Fc/F(ab)_2_ of the areas of the exposed Fc and F(ab)_2_ domains. The data points in Figure 4b, corresponding to the physisorbed IgG molecules, can be assigned to three groups (marked as gray rectangles) with distinct values of the mean PC3 scores, as confirmed by the ANOVA test. They reflect three different dominant antibody orientations as well as the changes, induced by increasing IgG surface density Γ, between flat-on, side-on and vertical (mixed head-on/tail-on) alignment. While flat-on orientation is expected for the lowest Γ values [11,12], a shift of the data points to higher PC3 score values is observed for the range 0.8 ≤ Γ ≤ 2.2 mg/m^2^ (Figure 4b), reflecting an adaptation of a side-on orientation for the IgG molecules. In turn, a shift of data points in the opposite direction is observed in Figure 4b for a further increase in surface density Γ (above 2.2 mg/m^2^). The level of PC3 scores attained by the data points is similar to that of IgG in flat-on orientation. This is interpreted as a transition to vertical antibody orientation with a mixed tail-on and head-on alignment, which reduces the ratio Fc/F(ab)_2_ to a value similar to that for flat-on orientation for comparable proportions of molecules with head-on and tail-on arrangements (Figure 4c). These proportions will be evaluated in the next section, where the analysis of surface density-dependent orientation of IgG molecules will be discussed for various immobilization methods.

### 2.3. Comparison of Surface Density Induced Orientation Changes of Antibodies Immobilized by Physical Adsorption and Two Covalent Coupling Methods

**Figure 5:**
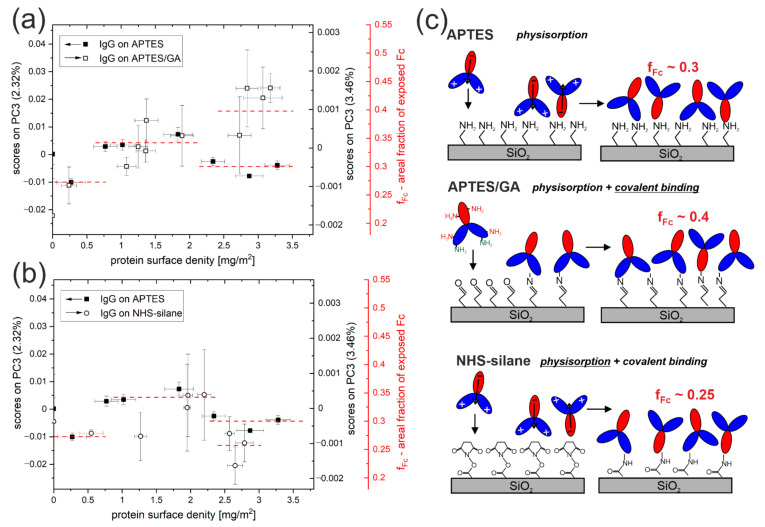
Comparison of the surface density-dependent orientation of IgG immobilized by physical adsorption on the APTES layer or by covalent coupling on the APTES/GA or NHS-silane layers [12]. Juxtaposition of the surface density dependence of the scores on the PC3 reflects changes in dominant IgG orientation for molecules physisorbed on APTES and covalently bound on APTES/GA (**a**) or NHS-silane modified surfaces (**b**). (**c**) Schematic presentation of the mechanism of immobilization of IgG molecules on examined silane layers by physical absorption or covalent attachment. Diagrams show the dominant IgG orientation revealed from TOF-SIMS studies for a high protein surface density corresponding to the vertical arrangement of molecules. The underlined terms mark the initial physisorption or covalent binding that is decisive for the final arrangement of antibodies.

The changes in the dominant antibody orientation with surface density examined for IgG molecules physisorbed on the APTES monolayer (Figure 4b) are compared with those determined [12] for the same molecules chemisorbed on APTES modified with glutaraldehyde (Figure 5a) or on an NHS-silane monolayer (Figure 5b). Both surface density Γ and IgG molecules orientation, represented by the scores on PC3, are determined in the same way. To compare the scores on PC3, obtained within individual PCA models, we apply the following approach: First, since the scores on Principal Components describing the TOF-SIMS data show linear correlation with the organic surface composition [29,30], an assumption is made about the linear relationship between the scores on PC3 and the composition of the outermost IgG region (i.e., examined with TOF-SIMS). The latter is described by the ratio *f_Fc_* of the exposed area of the Fc domain to the footprint area of the whole molecule in each particular orientation. Second, to allow comparison of the extent of changes in PC3 scores between different PCA models (i.e., different immobilization schemes), it is assumed [12] that the levels of PC3 scores for flat-on and side-on alignments correspond to *f_Fc_* values of 0.28 and 0.34, respectively, which are provided by MD simulations [16]. Under these assumptions, dominant antibody orientations are specified in terms of the *f_Fc_* values, marked in Figure 5a,b as an additional ordinate axis.

The overall behavior of the surface density Γ-dependent orientation of the IgG antibody (Figure 5a,b) physisorbed on amino-silane layers (solid squares) resembles those determined for the same molecule immobilized applying the two covalent coupling methods (open squares or circles). The transition to a more vertical orientation is expected every time the surface mass density Γ reaches a critical value (Γ_ind_Φ_∞_), which is specified by the packing efficiency Φ_∞_ and the mass loading Γ_ind_ of the individual molecule in a given orientation. In particular, for all the immobilization strategies, the Γ_ind_ values revealed by geometric considerations [11,16] produce critical values, 1.1 mg/m^2^ [11] to 1.4 mg/m^2^ [16] for flat-on, and ca. 1.9 mg/m^2^ [16] for side-on alignment, which are comparable with the observations only when random (Φ_∞_ ≈ 0.55 [15]) rather than close packing (Φ_∞_ ≈ 0.91) of IgG molecules is allowed.

However, a more detailed examination of Figure 5a,b also reveals differences between the various immobilization methods. The change from flat-on to side-on dominant alignment occurs at a lower Γ value (≈0.8 mg/m^2^) for IgG physisorption on the APTES monolayer than for chemisorption (≈1.2 mg/m^2^) either on APTES/GA (Figure 5a) or on the NHS-silane modified substrate (Figure 5b). In the course of protein adsorption on a surface, its density increases up to a maximum plateau value Γ determined by the adsorption isotherm. This process results in a reduction of the average area accessible to each molecule and increased repulsive intermolecular interactions, which force a more vertical molecular arrangement once a critical surface mass density value (Γ_ind_Φ _∞_) is reached. More vertical average orientation can be achieved due to IgG monolayer building mechanisms [10,31,32,33], such as orientational rearrangements of adsorbed proteins, the displacement of more horizontally aligned molecules in favor of more vertically ordered, and the filling of interstices between the more horizontally aligned proteins with the more vertically ordered ones. Most of these different mechanisms would be more effective for physisorption than covalent immobilization. Therefore, a transition to a more vertical dominant orientation is expected at lower equilibrium values Γ for antibodies on APTES rather than APTES/GA or NHS-silane surfaces, as it is can be concluded by the data presented in Figure 5a,b.

Different values of the areal fraction of exposed Fc domains are obtained for vertical alignment (Γ > 2.2 mg/m^2^) of antibodies immobilized following different approaches, with *f_Fc_* ≈ 0.3 for physical adsorption on the APTES layer and *f_Fc_* ≈ 0.25 and ≈0.4 for covalent coupling to NHS-silane and APTES/GA monolayers, respectively (Figure 5). *f_Fc_* values for IgG molecules on APTES and NHS-silane correspond to comparable proportions of IgG molecules with head-on and tail-on alignment: 4:3 for APTES and 1:1 for NHS-silane. On the contrary, a significantly different behavior is determined for antibodies on APTES modified with GA, where a proportion of 3:1 is determined. These findings confirm the conclusions of Section 2.1: GA activation of the APTES layer results in a higher fraction of molecules with head-on rather than tail-on alignment of adsorbed IgG molecules as the surface density increases (Figure 5c).

The differences in the specific vertical orientations attained by the IgG molecules on various silane monolayers can be explained in terms of molecule–surface and molecule–molecule interactions. The experimental conditions applied in the different immobilization procedures, in particular the use as an IgG solution of a buffer with pH 7.4, do not cause effective surface charging of APTES, APTES/GA, and NHS-silane monolayers [12]. In turn, electric dipoles are formed between the Fc and F(ab)_2_ domains at neutral pH [8]. Electrostatic interactions between antibody dipoles and the low-charged surface are not expected to affect our results, which are depicted schematically in Figure 5c. On the contrary, electrostatic dipole–dipole forces between proteins adsorbed with high surface density can promote the opposite orientation in neighboring dipoles [34]. Hence, the comparable proportions of head-on and tail-on orientation are adapted by the IgG molecules physisorbed on APTES silane. In turn, covalent binding of the antibody to aldehyde and NHS-silane can occur by reaction with ε-amine of lysine, which is randomly distributed in all antibody domains, and with a more reactive (due to lower pKa) α-amine of N-terminus located on the F(ab)_2_ domains. As a result, this covalent protein coupling promotes head-on orientation more than tail-on alignment, which competes with the opposite dipole orientations of neighboring antibodies characteristic for physisorption. Therefore, a mixed head-on and tail-on molecular arrangement concluded for IgG molecules immobilized on NHS-silane modified surface indicates effective intermolecular dipole–dipole interactions and the dominant role of physisorption that precedes covalent bonds formation [12]. In contrast, intermolecular forces, which are effective during physisorption, appear to be less decisive than covalent binding for the final arrangement of antibodies on a glutaraldehyde-activated APTES monolayer. The 3:1 proportion of IgG molecules with head-on to tail-on orientation correlates well with immobilization through the reactions with α-amine-groups of the N-terminus being more reactive than the ε-amine-groups of lysine residues [12,35]. The different impacts of intermolecular forces on the alignment of IgG molecules chemisorbed on NHS-silane and on APTES/GA-modified surfaces reflect the different roles of initial physical adsorption between these two surfaces compared to pure physisorption on APTES. This could be due to the faster immobilization of proteins on the APTES/GA surfaces that contain amine groups [12,14].

## 3. Materials and Methods

### 3.1. Silicon Substrates Functionalization and Proteins Immobilization

Before modification with 3-aminopropyltriethoxysilane (APTES, Sigma-Aldrich, Darmstadt, Germany), silicon substrates with native SiO_2_ layers (Si-Mat GmbH, Kaufering, Germany) were cleaned by subsequent sonication in toluene and ethanol for 10 min and by treatment in oxygen plasma for 30 s. Silanization was performed by immersion of the substrates in a 1% (*v*/*v*) APTES solution in toluene for 10 min, which is followed by washing with toluene and ethanol in an ultrasonic bath, drying under nitrogen stream and backing for 20 min at 120 °C [12]. Subsequent surface activation with aldehyde groups for APTES/GA samples was performed by the immersion of APTES-modified substrates in a 2.5% (*v*/*v*) aqueous glutaraldehyde solution (Sigma-Aldrich, Darmstadt, Germany) for 20 min, which was followed by washing with distilled water and drying under nitrogen stream. Polyclonal goat anti-rabbit IgG antibody (Thermo Fisher Scientific, Rockland, MA, USA), as well as F(ab)_2_ (Thermo Fisher Scientific, Rockland, MA, USA) and Fc (US Biological, Salem, MA, USA) domains of this antibody were immobilized on APTES and APTES/GA-modified substrates. To compare the dominant orientation of the antibodies on APTES before and after GA activation, the substrates were incubated with a 100 µL droplet of a 500 μg/mL solution of whole IgG, F(ab)_2_ domain, or Fc domain in PBS buffer (pH 7.4, 0.15 M, Sigma-Aldrich, Darmstadt, Germany) for 30 min. This concentration provides a high IgG surface density with Γ > 2.2 mg/m^2^ (see Figure S1 in Supplementary Materials). Furthermore, to determine the surface density-dependent orientation of antibodies on APTES-modified substrates, several samples were prepared similarly by incubation with IgG solutions with concentrations ranging from 5 μg/mL to 1 mg/mL. After incubation with the IgG solution, all samples were extensively washed with distilled water and dried under a nitrogen stream prior to examination with surface science techniques. The uniformity of the IgG layers was confirmed by AFM examination and TOF-SIMS imaging (see Section S3 in the Supplementary Materials). AFM images were recorded with an Agilent 5500 microscope working in non-contact mode. AFM probes with a spring constant of about 2 N/m, tip radius of about 7 nm and resonant frequencies of about 70 kHz were used.

### 3.2. Surface Density Determination with Spectroscopic Ellipsometry

The Sentech SE800 (Sentech Instruments GmbH, Berlin, Germany) Spectroscopic Ellipsometer was applied to determine the surface density of the antibodies. Measurements were performed over a wavelength range of 320–700 nm and at a fixed incidence angle equal to 70°. To estimate the average thickness of the protein layer, the obtained spectra were analyzed with SpectraRay 3 software and fitted by the Cauchy dispersion two-layer model consisting of the silicon substrate/mixed SiO_2_ and silane/protein layer. Fixed refractive index values equal to *n* = 3.87 for Si, *n* = 1.46 for SiO_2_, APTES and glutaraldehyde, and *n* = 1.53 for protein were used [12] The thickness of the native SiO_2_ layer and APTES layers determined for bare and silane-modified silicon substrates were equal to 2.7 ± 0.1 nm and 0.8 ± 0.1 nm, respectively, and they were taken as a constant to fit the thickness of the protein layer. The protein surface density Γ was estimated from the thickness d of the protein layer following the Cuypers one-component approach [12].

### 3.3. TOF-SIMS Characterization

TOF-SIMS analysis was performed with a TOF.SIMS 5 instrument (ION-TOF GmbH, Münster, Germany) equipped with a 30 keV liquid metal ion gun. Bi_3_^+^ ion clusters were applied as the primary beam, and a low energy electron flood gun was used for charge compensation. For all measurements, the ion dose was kept below 10^12^ ion/cm^2^ to ensure static mode conditions, and a 0.5 pA current was applied. High mass resolution TOF-SIMS spectra and maps of positive ions were recorded from ten non-overlapping 100 μm × 100 μm areas of each sample with resolution of 128 × 128 points. Spectra mass calibration was performed based on peaks of H^+^, H_2_^+^, CH^+^, C_2_H_2_^+^ and C_4_H_5_^+^. The mass resolution (m/Δm) was above 8000 at C_4_H_5_^+^ for all measurements.

### 3.4. Multivariate PCA Analysis of TOF-SIMS Data

Principal Component Analysis was performed using the PLS Toolbox (Eigenvector Research, Manson, WA, USA) for MATLAB (MathWorks, Inc., Natick, MA, USA). Before PCA was run, the intensities of selected peaks from each spectrum were normalized to the sum of selected peaks and mean-centered. The fluctuation of normalized TOF-SIMS signals derived from amino acids is below 10% for the spectra recorded in different TOF-SIMS operations.

## 4. Conclusions

The dominant orientation of IgG antibodies physically adsorbed on the APTES-modified silicon substrate was examined as a function of their surface density Γ (Figure 4): IgG orientation was characterized by Principal Component (PC) scores calculated by projection of TOF-SIMS spectrometry data on the developed PCA model, and Γ values were determined by ellipsometric spectroscopy. The results for antibodies physically adsorbed on amino-silane monolayers were compared with those [12] for antibodies covalently attached to glutaraldehyde-modified APTES (APTES/GA) and NHS-silane surfaces (Figure 5). The revealed Γ ranges for flat-on, side-on, and vertical orientation are consistent with random molecular packing. However, antibody reorientation occurs at lower Γ for physisorption than chemisorption, which is in accord with monolayer building models [10,31,32,33].

Furthermore, the immobilization method determines the vertical orientations of IgG molecules at high Γ, which is reflected by the values of the areal fraction *f_Fc_* of the exposed Fc domain. For APTES and NHS-silane, the *f_Fc_* values indicate comparable proportions of molecules adapting head-on and tail-on alignment, which are related to intermolecular dipole–dipole interactions. In turn, molecules immobilized on APTES/GA are characterized by a 3:1 proportion of head-on to tail-on orientation, which is linked with covalent binding. Moreover, the impact of glutaraldehyde activation of APTES, which is one of the most commonly applied methods for chemical activation of silicon surfaces [2,3], on the vertical orientations of adsorbed IgG molecules was confirmed by PCA analysis of separate TOF-SIMS measurements (Figure 2 and Figure 3).

Due to the high impact of the dominant antibody orientation on the biorecognition efficiency and bioassay performance, the presented approach to compare surface density-dependent antibody orientation for different immobilization methods can be applied to develop and optimize the protocols for biosensor functionalization.

## Data Availability

The data presented in this study are available on request from the corresponding author.

## Supplementary Materials

The following supporting information can be downloaded at: https://www.mdpi.com/article/10.3390/molecules27123672/s1, Section S1: Adsorption isotherm of IgG on APTES-modified substrate; Figure S1: Adsorption isotherm of goat anti-rabbit IgG on the SiO_2_ surface modified with APTES, APTES/GA and NHS-silane; Table S1: Binding capacity (BC) and affinity constant (AC), determined within the Langmuir and Random Sequential Adsorption model for adsorption data of goat anti-rabbit IgG on SiO_2_ surface modified with APTES, APTES/GA and NHS-silane; Section S2: Loading plots of the PCA model developed to examine surface density dependent orientation of IgG molecules adsorbed on APTES-modified substrate; Figure S2: TOF-SIMS and PCA analysis of the surface density-dependent orientation of IgG molecules on APTES-modified silicon substrates. PCA loadings plot for PC3 and for PC1; Section S3: Evaluation of IgG layers uniformity; Figure S3: Representative AFM topographic images of IgG molecules layers immobilized on SiO_2_ substrates functionalized with APTES with different surface density; Figure S4: Representative TOF-SIMS maps (Si^+^ ion from substrate and two protein derived ions C_4_H_8_N^+^ and C_5_H_12_N^+)^ recorded on layers of IgG molecules immobilized on SiO_2_ substrates functionalized with APTES with different surface density.

## Sample Availability

Samples are not available from the authors.
