# Supplementary material for: Comparison of Physical Adsorption and Covalent Coupling Methods for Surface Density-Dependent Orientation of Antibody on Silicon
Source: Molecules. 2022 Jun 7;27(12):3672. doi: 10.3390/molecules27123672 (PMC9228713; doi:10.3390/molecules27123672)
Supplement: Supplementary file 1 [file molecules-27-03672-s001.zip › molecules-1721700-supplementary.pdf]

molecules

Supplementary Materials

Comparison of Physical Adsorption and Covalent Coupling Methods for Surface Density-Dependent Orientation of Antibody on Silicon

Katarzyna Gajos [1,*], Panagiota Petrou [2] and Andrzej Budkowski [1]

[1] Smoluchowski Institute of Physics, Jagiellonian University, Łojasiewicza 11, 30-348 Kraków, Poland; katarzyna.gajos@uj.edu.pl (K.G.); andrzej.budkowski@uj.edu.pl (A.B.)
[2] Institute of Nuclear & Radiological Sciences & Technology, Energy & Safety, NCSR Demokritos, 15341 Aghia Paraskevi , Athens, Greece; ypetrou@rrp.demokritos.gr
* Correspondence: katarzyna.gajos@uj.edu.pl

S1. Adsorption isotherm of IgG on APTES-modified substrate

Surface density values Γ of IgG antibody, immobilized with various concentrations *c* of IgG solutions, and determined for SiO_2 substrates modified with APTES (this study, solid squares) are compared with similar results obtained earlier [8] for APTES/GA (open diamonds) and NHS-silanes [8] (open squares) in Fig. S1. The data are described using Langmuir adsorption model with solid lines that are a guide to eye. In addition, description provided by Random Sequential Adsorption (RSA) model for APTES-modified substrate is shown as a dashed line. The data can be described with two adsorption parameters: binding capacity (BC) and affinity constant (AC), provided by both Langmuir (eq. S1) and RSA model (eq. S2) [8]:

$$\Gamma = BC\frac{c}{c+1/AC} \tag{S1}$$

$$c = \frac{\Gamma}{BC}(1/AC)/\left[\left(1+0.812\frac{\Gamma}{BC}+0.4258\left(\frac{\Gamma}{BC}\right)^2+0.0716\left(\frac{\Gamma}{BC}\right)^3\right)\left(1-\frac{\Gamma}{BC}\right)^3\right] \tag{S2}$$

The values of adsorption parameters, determined within both models for IgG adsorbed on APTES-modified substrate, are compared in Table S1 with the values obtained previously [8] for APTES/GA and NHS-silanes.

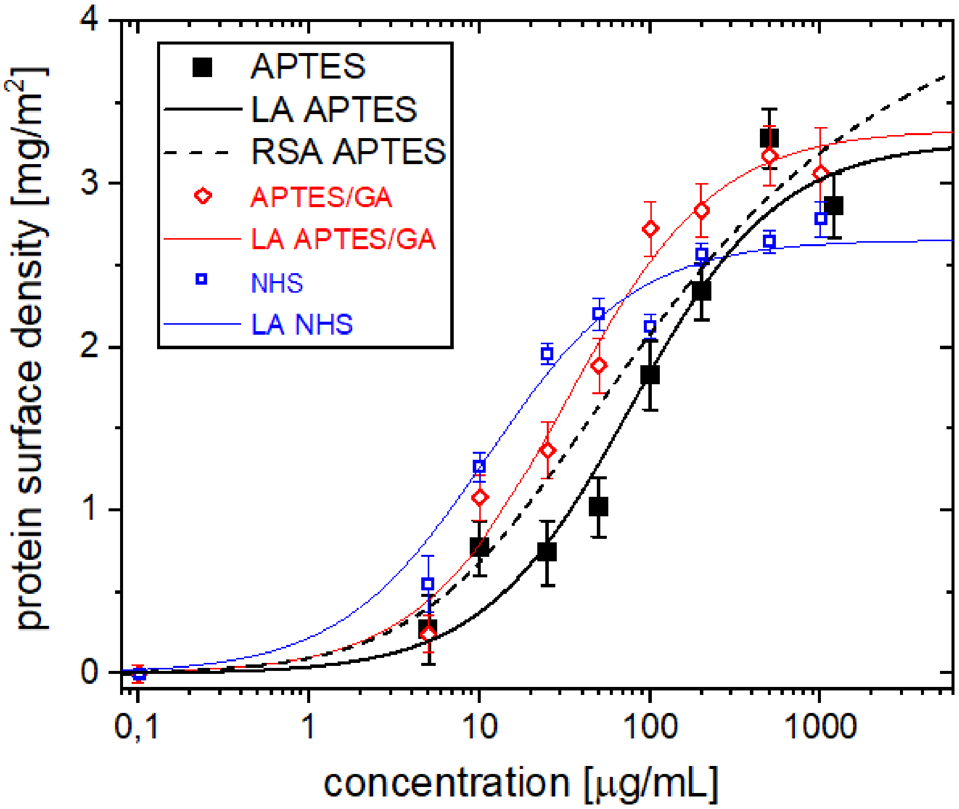

Figure S1. Adsorption isotherm of goat anti-rabbit IgG on SiO_2 surface modified with APTES (this study, solid squares), APTES/GA [8] (open diamonds) and NHS-silane [8] (open squares). Solid lines are a guide to eye and describe the experimental data on basis of Langmuir model (LA). In addition, the description provided by Random Sequential Adsorption (RSA) model for APTES-modified surface is marked as a dashed line.

Table S1. Binding capacity (BC) and affinity constant (AC), determined within Langmuir and Random Sequential Adsorption model for adsorption data of goat anti-rabbit IgG on SiO_2 surface modified with APTES (this study), APTES/GA [8] and NHS-silane [8].

	Langmuir model		RSA model	
	**BC [mg/m²]**	**AC [x 10⁷ 1/M]**	**BC [mg/m²]**	**AC [x 10⁷ 1/M]**
APTES	~3.3	~0.2	~4.3	~0.4
APTES/GA	~3.3	~0.5	~3.8	~1.6
NHS	~2.7	~1.4	~3.2	~2.8

S2. Loading plots of the PCA model developed to examine surface density dependent orientation of IgG molecules adsorbed on APTES-modified substrate

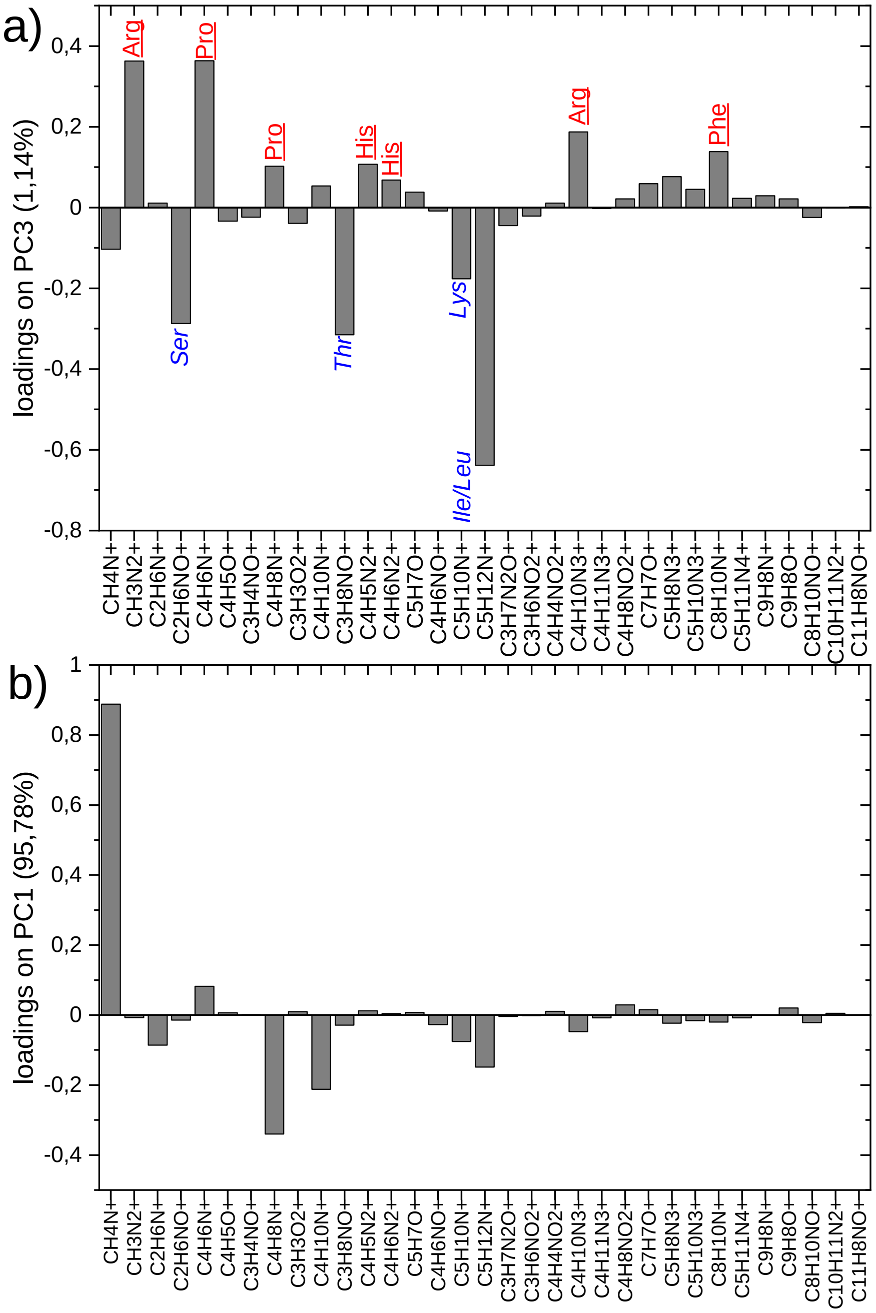

Figure S2. TOF-SIMS and PCA analysis of the IgG molecules surface density dependent orientation on APTES modified silicon substrates. PCA loadings plot for the PC3 (a) and for the PC1 (b).

S3. Evaluation of IgG layers uniformity

To evaluate uniformity of IgG layers topographic AFM images were recorded with an Agilent 5500 microscope working in non-contact mode. AFM probes with spring constant about 2 N/m, resonant frequencies about 70 kHz and tip radius below 7nm were used. Several AFM images were taken at different areas for each sample.

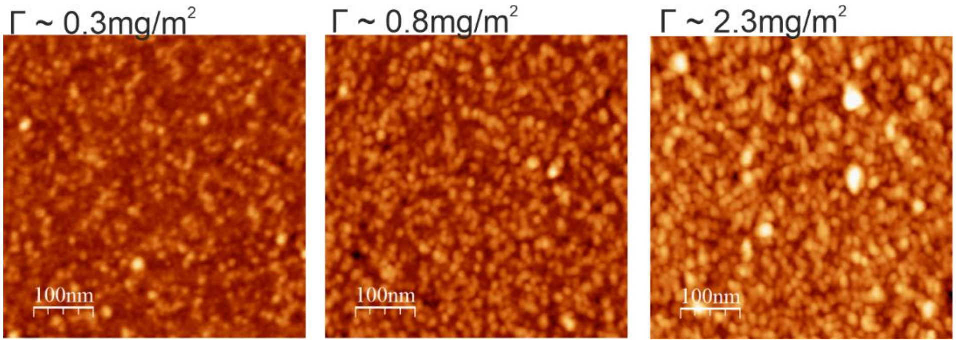

Figure S3. Representative AFM topographic images of IgG molecules layers immobilized on SiO_2 substrates functionalized with APTES with different surface density. AFM analysis confirms uniformity of IgG layers.

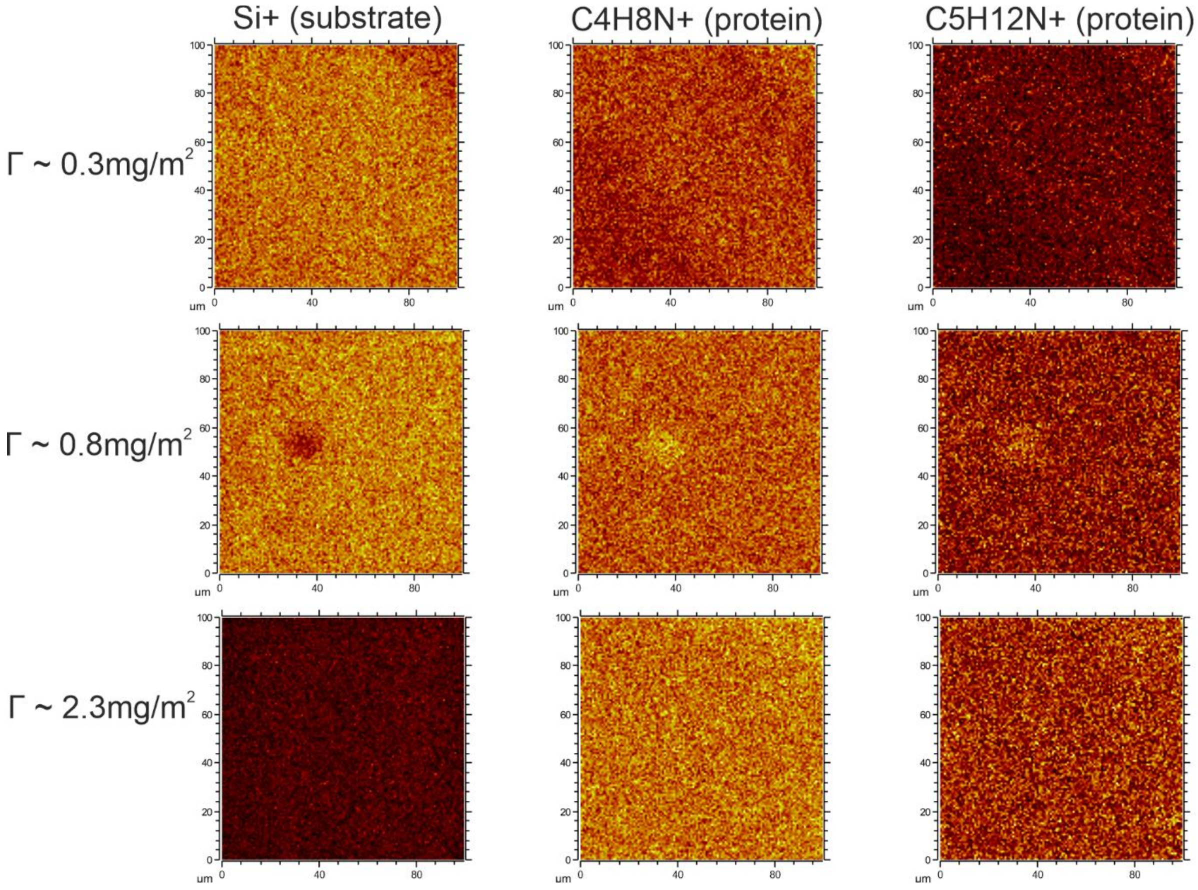

Figure S4. Representative TOF-SIMS maps (Si^+ ion from substrate as well as two protein derived ions $C_4H_8N^+$ and $C_5H_{12}N^+$) recorded on IgG molecules layers immobilized on SiO_2 substrates functionalized with APTES with different surface density. TOF-SIMS imaging confirms good uniformity of IgG layers.
